# A Novel Technique for Bulk-Fill Resin-Based Restorations: Achieving Function and Esthetics in Posterior Teeth

**DOI:** 10.1155/2017/9408591

**Published:** 2017-11-26

**Authors:** Gerardo Durán Ojeda, Ismael Henríquez Gutiérrez, José Pablo Tisi, Abelardo Báez Rosales

**Affiliations:** ^1^Facultad de Ciencias de la Salud, Universidad Arturo Prat, Iquique, Chile; ^2^Restorative Dentistry Department, University Andrés Bello, Viña del Mar, Chile

## Abstract

Advances in the mechanical properties of composite resins have allowed for their use in posterior teeth. Conventional resins have several problems associated with polymerization shrinkage stress. The development of “bulk-fill” resins has allowed for their use in single increments up to depths of 4 mm, with very low polymerization shrinkage stress. Nevertheless, differences in anatomy and the desire for optimal esthetics present unique difficulties. This article describes a step-by-step technique using flowable bulk-fill resin as a substitute for dentin in a single increment, together with a high-reflective-index resin to restore enamel and decrease clinical time, obtaining anatomically and esthetically acceptable results without detriment to the mechanical properties required to restore the functionality of the posterior teeth.

## 1. Introduction

Posterior teeth represent a scenario of variable complexity when clinicians are performing a direct or indirect restoration using adhesive-resin-based materials. The application of conventional resins using the stratification technique is associated with the risk of incorporating bubbles and impurities, longer clinical time, and polymerization shrinkage stress [1–3]. Additionally, subsequent problems arising from polymerization shrinkage stress can occur, such as microleakage at the margins [1], cuspal deflection and enamel cracks [4–6], an increased predisposition to the formation of secondary caries, and postoperative sensitivity [7, 8].

Despite issues with the stratification technique in the posterior teeth, the longevity of these restorations has been examined in previous reports with high rates of clinical success based on randomized and retrospective longitudinal studies, with results showing survival times of up to 18–20 years [9–12].

Given the problems associated with polymerization shrinkage stress, new composite resins, termed “bulk-fill” resins, are now available on the dental market for use in single-increment applications up to 4–5 mm [13]. These resins initially had a flowable consistency for use as base materials or liners, which are then complemented with a final layer of a conventional composite resin. The first of these materials was SureFil® SDR® flow (DENTSPLY, Konstanz, Germany), which used “stress decreasing resin” (SDR) technology and provided greater flexibility, allowing for dissipation of stress during the polymerization reaction [14].

The present case report details a step-by-step procedure to restore two molars with old failed amalgam restorations classified as “Charlie” according to the Modified United States Public Health Service (USPHS) Ryge Criteria for Direct Clinical Evaluation of Restoration, which presented color mismatch, marginal discoloration, loss of restorative substance in the anatomic contour, microleakage, and secondary caries. This led to the decision to restore with a new restoration instead to repair both amalgams and the remaining tooth structure. The technique of choice in this case includes a mix of a flowable bulk-fill resin, with the anatomical completion of the enamel using a conventional restorative resin. An 18-month follow-up allowed for evaluation of the results.

## 2. Case Report

**Figure 1:**
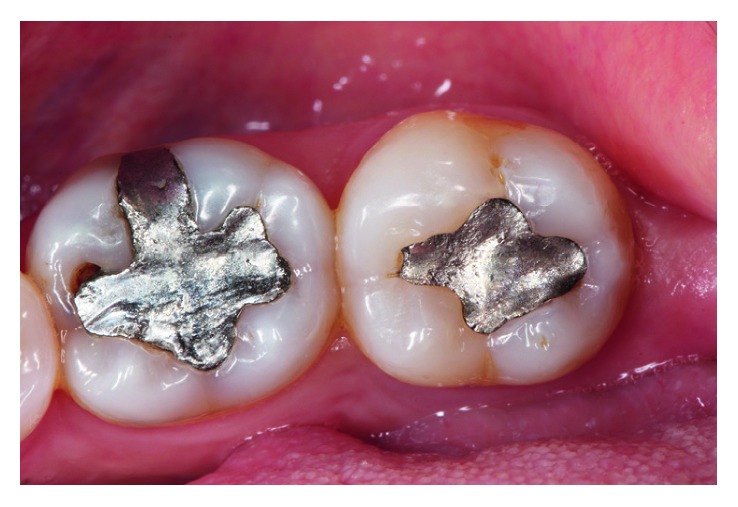
Failed OV amalgam filling in tooth 36 and an old occlusal amalgam restoration in tooth 37.

A 52-year-old female patient presented to the private practice of one of the authors with a main complaint of two old failed amalgam restorations in teeth 36 (OV) and 37 (O), requesting that these restorations be replaced with new composite resins. After a complete clinical examination, it was determined that both teeth were vital (Figure 1).

**Figure 2:**
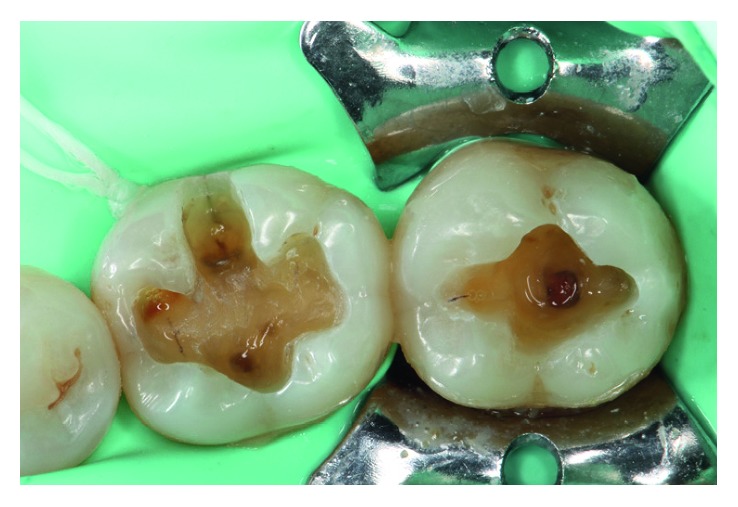
Elimination of the failed 36 OV amalgam and the occlusal amalgam in tooth 37 under rubber dam isolation.

**Figure 3:**
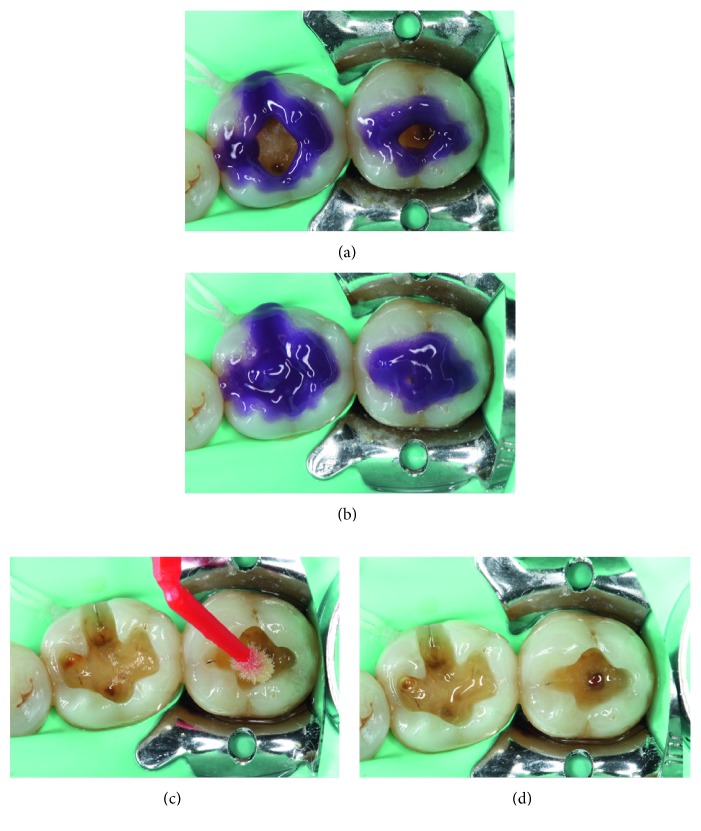
Adhesive procedure. (a) Application of 37.5% phosphoric acid for 15 sec in enamel. (b) The dentin is now being etched for 15 sec. (c) A thin layer of primer is being applied. (d) Glossy aspect of the bonding layer once the technique is finished.

Under rubber dam isolation, the elimination of the old amalgams was performed with carbide burs (H4MCL.314.012, Komet, Gebr. Brasseler GmbH & Co., Germany), attempting to spare a healthier dental structure (Figure 2). Once the tooth preparations were completed, the adhesive technique was performed on both teeth in the same manner. Initially, the enamel was etched with 37.5% phosphoric acid (Gel Etchant, Kerr, Orange, CA, USA) for 15 sec, after which the dentin was etched for 15 sec. The acid was then rinsed off with an air/water spray for 30 sec and air-dried, taking care not to desiccate the dentin. After the tooth surface had been treated, a first layer of primer was applied to the dentin and rubbed using a microbrush for 20 sec (Primer, OptiBond FL, Kerr, Orange, CA, USA). For the enamel, primer was applied gently without rubbing. An air jet allowed for primer runoff and solvent volatilization, after which a thin layer of bonding was applied and light-cured for 20 sec (Bonding, OptiBond FL, Kerr, Orange, CA, USA) with a light-emitting diode (LED) curing light unit (Coltolux® LED, Coltene/Whaledent Inc., OH, USA) (Figure 3).

**Figure 4:**
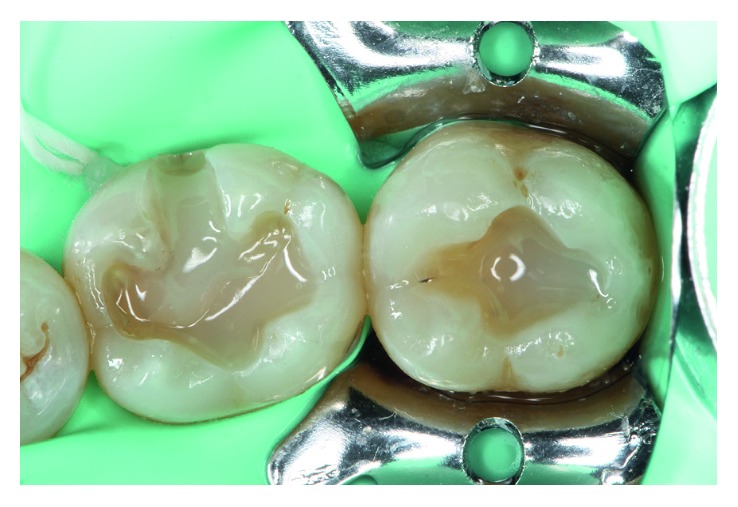
Flowable bulk-fill resin has been applied (SureFil SDR flow, DENTSPLY, Konstanz, Germany).

A layer of flowable bulk-fill resin (SureFil SDR flow, DENTSPLY, Konstanz, Germany) was then applied at the base surface of both tooth preparations in single-layer increments until the dentin was completely full, leaving sufficient space (approximately 1 mm) to apply an enamel resin material (Figure 4). Once this layer was placed, it was polymerized for 20 sec.

**Figure 5:**
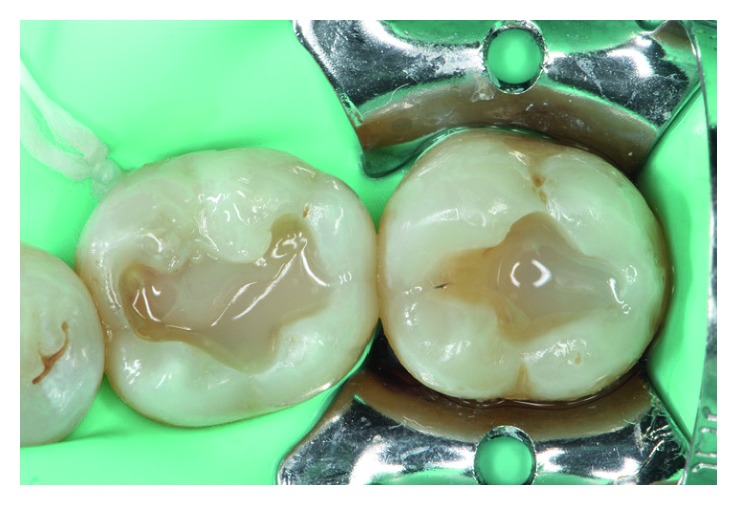
Vestibular wall of tooth 36 has been restored with a layer of conventional high-refractive-index resin (UE1, Micerium S.P.A., Avegno, GE, Italy).

**Figure 6:**
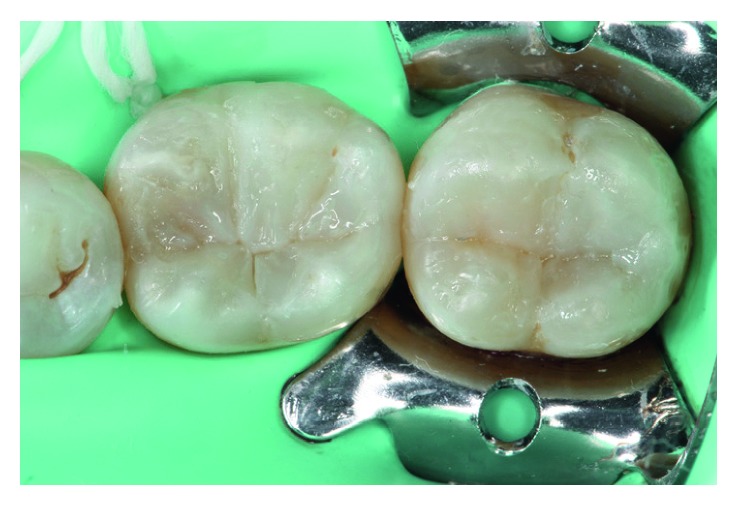
The complete enamel has been restored with a conventional composite resin. Notice the correct anatomy achieved in both teeth.

**Figure 7:**
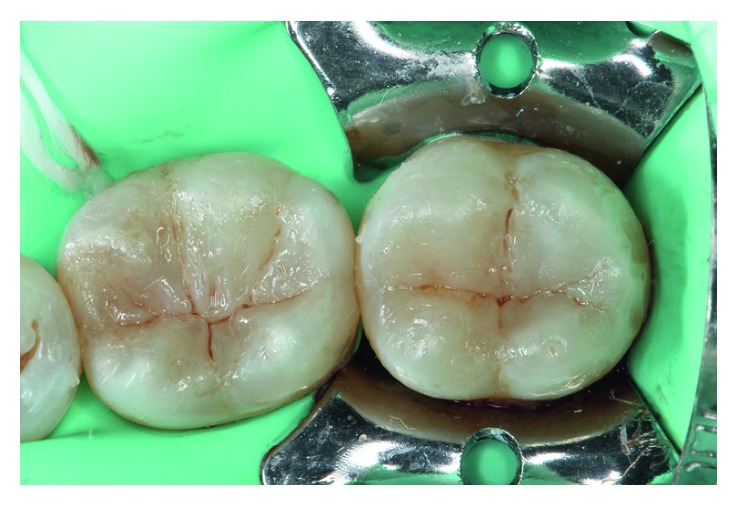
Resin pigments were used to characterize the depths of pits and fissures.

To complete the occlusal morphology of both teeth, a conventional stratification resin was selected (UE1, ENA HRi, Micerium S.P.A., Avegno, GE, Italy) that was applied in single-layer increments to complete the final anatomy of the lost enamel, after which it was light-cured for 40 sec (Figures 5 and 6). To characterize these teeth, flowable resin pigmentations were applied (Brown2, Micerium S.P.A., Avegno, GE, Italy) (Figure 7). A final polymerization through a layer of glycerin was performed for 40 sec for each tooth to eliminate the polymerization inhibition layer.

**Figure 8:**
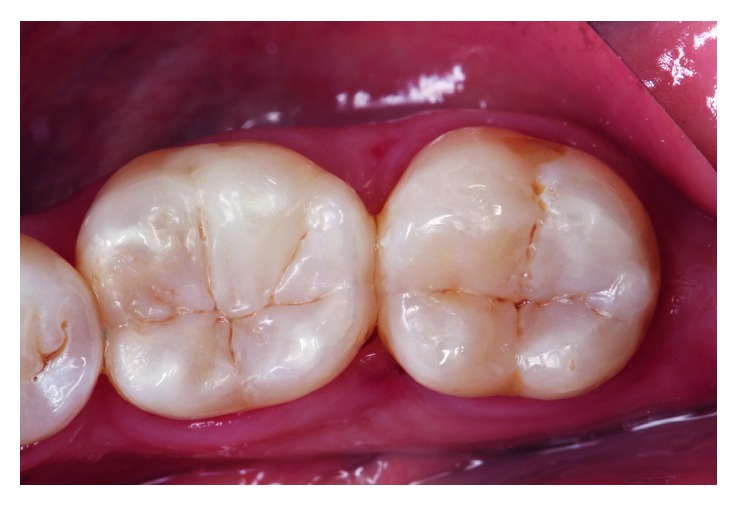
Immediate result after polishing and finishing.

**Figure 9:**
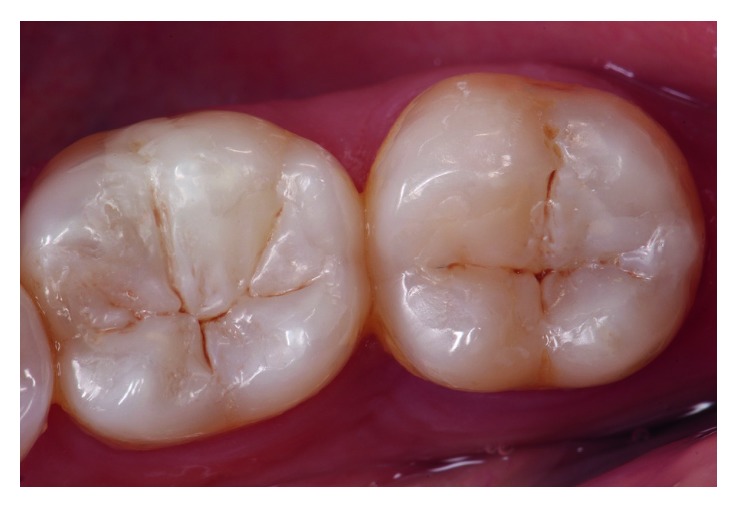
Image taken at the 18-month follow-up.

Finally, finishing and polishing were performed. In this case, the procedure started with Enhance diamond points (Enhance®, DENTSPLY, Konstanz, Germany) for prepolishing, together with diamond polishing pastes (Shiny A and Shiny B, Micerium S.P.A., Avegno, GE, Italy) and a final aluminum oxide paste (Shiny C, Micerium S.P.A., Avegno, GE, Italy). An immediate control image is shown in Figure 8 and 18-month follow-up in Figure 9.

## 3. Discussion

This article describes a reduced-step approach for exchanging amalgam restorations with composite resins using a base of flowable bulk-fill resin as a dentin substitute in combination with a final layer of conventional composite resin to replace the enamel and achieve anatomically and esthetically acceptable results.

Dental amalgam is commonly used as a restorative material to restore posterior teeth. At this time, its use is limited for various reasons, namely, it has been associated with cracked tooth syndrome [15–17], its physical-mechanical properties differ from those of natural dental structures, the esthetics are poor, less conservative tooth preparations may be needed due to their lack of adhesiveness [18], and there is a potential release of mercury, which can be toxic [19, 20].

Owing to these disadvantages and advances in the mechanical properties of composite resins, novel restorative materials have been optimized for restoration of the posterior region. The first materials used for the posterior region included conventional composite resins, whose volumetric shrinkage after polymerization ranges between 1.35% and 7.1% [21], which generated cuspal deflection [4, 6, 22, 23], thereby increasing the probability of enamel microcrack formation, showing unavoidable adhesive failure of the tooth-restoration interface [24], and leading to microleakage formation [25].

Flowable bulk-fill resins decrease polymerization shrinkage stress and have a better degree of conversion than restorative bulk-fill resins at depths of 4 mm [26]. The flowable bulk-fill material used in this case (SureFil SDR flow, DENTSPLY, Konstanz, Germany) showed greater depth of cure and a higher degree of conversion than similar flowable resins, conventional flowable composites, and restorative bulk-fill composites [27]. This resin has superior mechanical properties compared to conventional flowable composites because of lack of the mechanical properties of high-density conventional restorative resins, justifying its incorporation into the final layer of incremental technique resins to protect the fluid material from potential wear [28, 29].

According to Hirata et al. [30], there are two clinical approaches for using bulk-fill materials to restore posterior teeth. The first is to use restorative bulk-fill material (high density) in a single increment in cavity preparations up to 4 mm deep, and the second is to apply a bulk-fill flowable resin as a base material for dentin replacement in a single increment, finished with a final layer of conventional composite resin to restore the enamel. We found that a relatively inexperienced clinician may find the first technique difficult to perform, as the carving procedure must be executed quite quickly. In addition, this technique may result in less color stability over time compared with conventional resin systems [31, 32], affecting the final esthetic result.

Advantages of the presented technique include a greater stability of color because the final enamel layer consists of a conventional resin that modifies the dental value (ENA HRi, Micerium S.P.A., Avegno, GE, Italy) and a high refractive index, identical to natural enamel (IR = 1.62) [33, 34]. These characteristics optimize appearance, leading to a more esthetic restoration, as well as decreasing the clinical restorative time. Fewer layers are required compared with the classical stratification technique for the posterior region, thus decreasing the clinical steps and making the technique less sensitive to the incorporation of bubbles between layers.

## 4. Conclusion

Based on our results, we recommend the use of flowable bulk-fill resins as a dentin substitute, finishing the enamel with a conventional high-refractive-index resin to improve the mechanical properties and optimize esthetics when performing restorations in posterior teeth.
